# Voice disorders in severe obstructive sleep apnea patients and comparison of two acoustic analysis software programs: MDVP and Praat

**DOI:** 10.1007/s11325-020-02102-4

**Published:** 2020-06-25

**Authors:** Mei Wei, Jianqun Du, Xiaoyu Wang, Honghua Lu, Wei Wang, Peng Lin

**Affiliations:** 1 Department of Otorhinolaryngology Head and Neck Surgery, Tianjin First Central Hospital, No. 24 Fukang Road, Nankai District, Tianjin, 300192 China; 2 Institute of Otolaryngology of Tianjin, Tianjin, China; 3 Key Laboratory of Auditory Speech and Balance Medicine, Tianjin, China; 4 Key Clinical Discipline of Tianjin (Otolaryngology), Tianjin, China; 5 Otolaryngology Clinical Quality Control Centre, Tianjin, China

**Keywords:** OSAHS, Acoustic analysis, MDVP, Praat, EGG

## Abstract

**Objective:**

The purposes of this study were to explore the effect of obstructive sleep apnea-hypopnea syndrome (OSAHS) on the voice by analyzing the acoustic parameters between patients with OSAHS and those without OSAHS and to compare acoustic analyses performed by two software programs (MDVP and Praat).

**Methods:**

Patients with OSAHS (n = 75) and normal controls (n = 46) were asked to produce a sustained sound of the vowel /i/ and were analyzed with electroglottography (EGG), MDVP, and Praat software. A self-rated scale (Voice Handicap Index, VHI-10) and acoustic parameters were compared.

**Results:**

There were no statistically significant differences in the fundamental frequency (F0), jitter, shimmer, noise/harmonic ratio (NHR), contact quotient perturbation (CQP), or contact index perturbation (CIP) between the patient group and the normal group. The VHI-10 values were significantly increased in patients with OSAHS. The receiver operating characteristic (ROC) analysis suggested that the shimmer obtained from MDVP and Praat possessed relatively high accuracy in differentiating patients with OSAHS from healthy individuals. The results for F0, jitter, shimmer, and NHR were significantly different between MDVP and Praat in OSAHS patients. In normal persons, there was a significant difference in NHR; however, no significant differences were found for F0, jitter, or shimmer between the two software programs. The results demonstrated that high correlations were found between values obtained by both software programs.

**Conclusions:**

Patients with OSAHS were prone to vibration irregularity, incomplete glottal closure, hoarseness, and other vocal problems. The two acoustic software programs present different values of acoustic measures. There was a strong correlation and consistency between the parameters calculated by the two software programs.

## Introduction

Obstructive sleep apnea-hypopnea syndrome (OSAHS) is characterized by the narrowing and collapse of pharyngeal structures during sleep, resulting in intermittent hypoxemia and sleep disruption. One or more sites of obstruction may include the soft palate, tongue, lateral walls, and/or epiglottis [1]. OSAHS has been identified as a risk factor for hypertension, heart disease, stroke, and even cancer [2–7]. This disease affects approximately 4 to 7% of adults in the general population [8]. In addition to repetitive interruptions in breathing during sleep, patients may have some signs and symptoms, including chronic snoring, memory or concentration problems, daytime sleepiness, sore throat, or dry mouth [9].

OSAHS patients have structural abnormalities in the upper airway (upper airway inflammation, thickened pharyngeal walls, hypertrophic tonsils, or a thickened and slack, soft palate), which lead to structural changes, and these changes affect voice production and resonance and contribute to abnormal voice features [10, 11]. It is possible that dryness and inflammation in the upper respiratory system may be caused by chronic snoring, which may adversely affect the health of the vocal folds and lead to disturbances in phonation. Recently, a study indicated that individuals who snore for a long time showed degraded voice quality compared with the voice quality of those who do not snore [12].

In this study, we focused on acoustic analysis software to assess OSAHS patients’ voice disorders by comparing the results of the acoustic analyses performed by two software programs: MDVP (Kay Elemetrics) and Praat (Boersma and Weenink). The two software programs are commonly used for acoustic analysis in clinical and research settings, while MDVP is the most used and cited acoustic analysis software [13]. Praat is distributed for free use and supported by many clinicians and scientists. The acoustic analysis software was used to objectively define F0, jitter, shimmer, and the noise-to-harmonics ratio (NHR). EGG can be used to monitor the vibrational patterns of the vocal folds. The advantage of EGG in the evaluation of the glottal wave was that it captures vibrations directly above the thyroid cartilage, without interference of the supraglottal activity or background noise. Contact index perturbation (CIP) and contact quotient perturbation (CQP) were obtained by EGG.

The aim of this study was to assess acoustic features of voice in OSAHS patients and to identify whether the results obtained from the same voice samples are comparable and/or correlative between the MDVP and Praat acoustic computer programs.

## Patients and methods

### Patients

This was a prospective study performed at Tianjin First Central Hospital. Two groups of subjects were recruited. The patient group consisted of 75 patients who were diagnosed with severe OSAHS (apnea-hypopnea index > 30/h) [14] by polysomnographic examination. According to the imaging examination, all the patients were obstructed by the pharynx and pharyngeal obstruction. The appearance and activity of the vocal cords under stroboscopic laryngoscopy were normal. The normal group included 46 healthy people with no symptoms, such as excessive daytime sleepiness, loud snoring, observed episodes of breathing cessation during sleep, abrupt awakenings accompanied by shortness of breath, and awakening with dry mouth or sore throat. The STOP-Bang questionnaire was used to screen for OSAHS in the normal group. The STOP-Bang questionnaire is a validated OSAHS screening tool. The questionnaire has 8 items, and each item is worth 1 point. If the score of the first 4 items added is ≥ 2 points, the risk of OSAS is considered to be high; if the STOP-Bang questionnaire is used completely, and the total score is ≥ 3, the risk of OSAS is considered to be high. The participants in the normal control group needed to complete 8 items and score no more than 3 points. The people in the two groups were male and had similar age and BMI distributions. Exclusion criteria for both the patient and normal group were smoking, comorbid diseases likely to affect the voice, using medications, history of laryngeal problems, Sjogren’s syndrome, pulmonary or neurologic disease, and other autoimmune disorders likely to change voice production.

### Acoustic voice analysis

Each voice sample was recorded while the examiner was under identical conditions in a sound-treated room with ambient noise below 50 dB. The person took a seat in front of the microphone placed 15 cm from their mouths. To reduce intrasubject variability, 5 samples of sustained vowel /i/ sounds at a comfortable pitch, constant amplitude, and flat tone were used for each subject. All voice samples were obtained using EGG and Speech Lab Model 4500 (Kay Elemetrics Corporation) at a sampling rate of 50 kHz and 16-bit quantization. Acoustic voice analysis was performed twice: once using MDVP and once using Praat. The fundamental frequency (F0), jitter, shimmer, and NHR were compared between MDVP and Praat. Additionally, CQP and CIP were obtained by EGG.

The Turkish version of the Voice Handicap Index-10 (VHI-10) was used to evaluate voice quality. The VHI-10 is a questionnaire that assesses a patient’s perception of the physical, emotional, and functional aspects of their voice [15]. Subjects receive a score of 0–4 for each question. The higher the score, the greater the degree of handicap.

In the present study, all acoustic voice parameters were evaluated in the morning, which prevented the voices of the participants from being affected by circadian changes [16].

### Statistical analysis

Statistical analysis of the data was performed using IBM SPSS statistics 19. The Mann-Whitney *U* test was used to compare the four markers between normal persons and patients. The receiver operating characteristics (ROC) approach was also applied to evaluate the diagnostic value of voice parameters in OSAHS patients, and the results were estimated by calculating the area under the ROC curve (AUC), sensitivity, and specificity according to standard formulas. Spearman’s rank correlation coefficient (Spearman’s rho) and Bland-Altman analysis were performed to assess the correlation and consistency of F0, jitter, shimmer, and NHR between the two acoustic voice programs: MDVP and Praat.

## Results

### Acoustic characteristics of OSAHS patients

The results of the acoustic analysis performed using the two software programs in normal persons and OSAHS patients are shown in Table 1. Statistical analysis revealed that there was no statistically significant difference between the normal group and the patient group in F0 according to the two analysis programs.

**Table 1:** The results of VHI-10 and comparison of acoustic parameters between OSAHS patients and normal persons

Parameter	Software	OSAHS	Normal	Cohen’s *d*
F0	MDVP	130.774 ± 26.367	139.812 ± 26.713	0.268
	Praat	128.455 ± 25.498	135.553 ± 23.468	0.338
Jitter	MDVP*	1.489 ± 0.822	0.541 ± 0.352	1.383
	Praat*	0.945 ± 0.602	0.522 ± 0.267	0.840
Shimmer	MDVP*	4.189 ± 1.571	2.683 ± 0.624	1.159
	Praat*	5.074 ± 1.96	2.860 ± 0.792	1.358
NHR	MDVP*	0.147 ± 0.029	0.123 ± 0.016	0.975
	Praat*	0.047 ± 0.259	0.037 ± 0.052	0.257
VHI*		3.09 ± 2.98	1.87 ± 2.08	0.453
CQP*	EGG	6.12 ± 6.11	2.19 ± 2.14	0.778
CIP*	EGG	42.83 ± 47.82	17.52 ± 13.80	0.272

*Indicates a significant difference

**Fig. 1:**
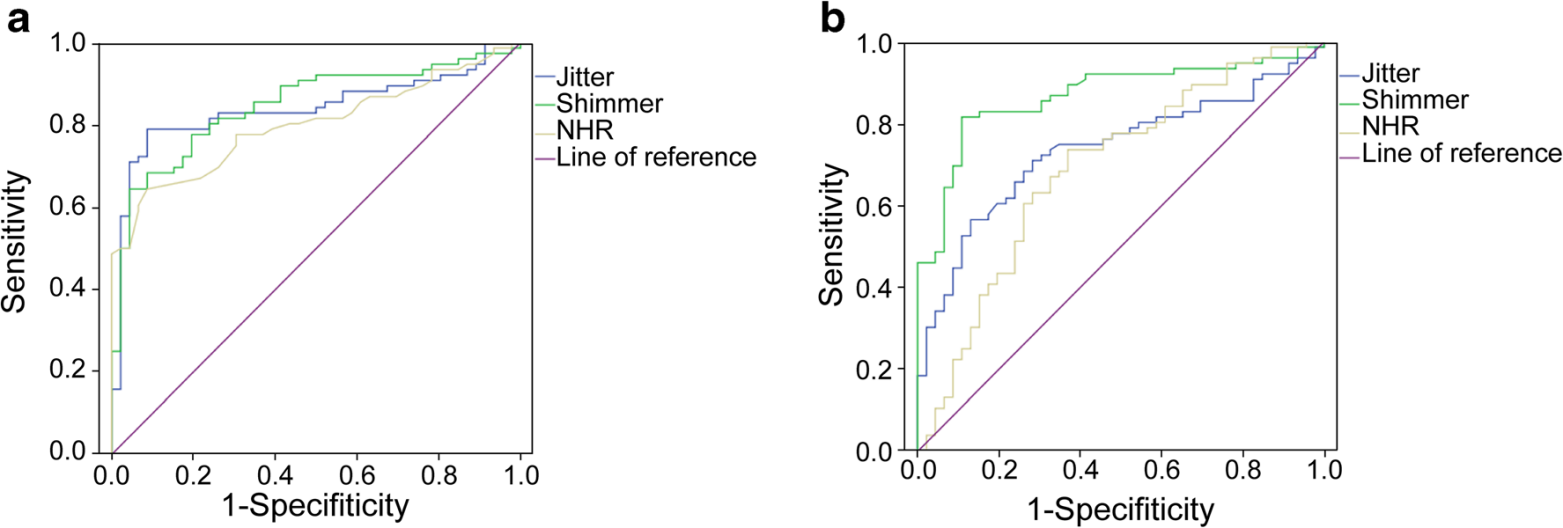
**a** ROC curves were established to evaluate the clinical value of jitter, shimmer, and NHR from MDVP in OSAHS patients. **b** ROC curves were established to evaluate the clinical value of jitter, shimmer, and NHR from Praat in OSAHS patients

In the MDVP analysis, there were significant differences in jitter, shimmer, and NHR between the two groups. The ROC curve analysis is shown in Fig. 1a. The AUC of jitter was 0.840 (95% CI 0.767–0.914), and the cutoff point was 0.774, with a sensitivity of 78.9% and specificity of 91.3%. The AUC of shimmer was 0.847 (95% CI 0.777–0.916), and the cutoff point was 3.598, with a sensitivity of 64.5% and specificity of 95.7%. The AUC of NHR was 0.799 (95% CI 0.723–0.876), and the cutoff value was 0.141, with a sensitivity of 65.5% and specificity of 91.3%. The results showed that shimmer assessed by MDVP possessed relatively high accuracy in differentiating OSAHS patients from healthy individuals.

For Praat, significant differences were also found in jitter, shimmer, and NHR between the normal group and patient group. The ROC curve analysis showed that the AUC of jitter was 0.736 (95% CI 0.648–0.825), and the cutoff was 0.568, with a sensitivity of 71.05% and specificity of 71.7%. The AUC of shimmer was 0.869 (0.804–0.934), and the cutoff was 3.47, with a sensitivity of 81.5% and specificity of 80.1%. Shimmer possessed relatively high accuracy in differentiating OSAHS patients from healthy individuals. The AUC of NHR was 0.689 (0.590–0.789), and the cutoff value was 0.035, with a sensitivity of 73.6% and specificity of 63.0% (Fig. 1b).

VHI-10 scores and acoustic parameters from EGG are also depicted in Table 1. The mean VHI-10 score of OSAHS patients was significantly higher than that of normal persons. In the OSAHS group, CQP and CIP for the EGG signals showed significantly greater values than those in the normal group (*P* < 0.001).

### The differences and correlations between MDVP and Praat

The results of the acoustic analysis performed using the two software programs for the two study groups are presented in Table 2. In addition to the shimmer, the values obtained for F0, jitter, and NHR were higher for MDVP than those obtained using Praat.

**Table 2:** Comparison of acoustic parameters obtained by MDVP and Praat

Parameter	Software	MDVP	Praat	Cohen’s *d*
F0	OSAHS^#^	130.774 ± 26.367	128.455 ± 25.498	0.169
	Normal	139.812 ± 26.713	135.553 ± 23.468	0.184
Jitter	OSAHS^#^	1.489 ± 0.822	0.945 ± 0.602	0.754
	Normal	0.541 ± 0.352	0.522 ± 0.267	0.062
Shimmer	OSAHS^#^	4.189 ± 1.571	5.074 ± 1.96	0.498
	Normal	2.683 ± 0.624	2.860 ± 0.792	0.255
NHR	OSAHS^#^	0.147 ± 0.029	0.047 ± 0.259	3.653
	Normal**	0.123 ± 0.016	0.037 ± 0.052	2.222

**The differences between the two programs in normal voice *P* < 0.05. ^#^The differences between the two programs in OSAHS patients *P* < 0.05

**Fig. 2:**
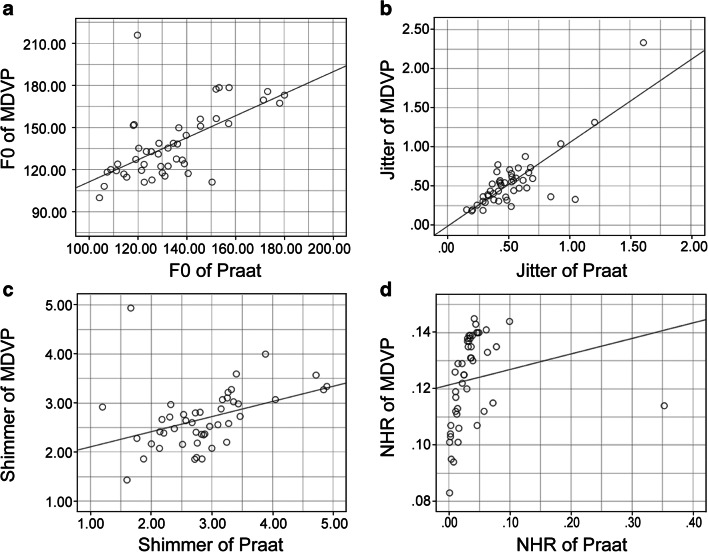
Normal voice data. The horizontal axis indicates the Praat data. The vertical axis indicates the multidimensional voice program (MDVP). **a** Scatter plot of the fundamental frequency. Spearman’s rho = 0.604. **b** Scatter plot of jitter. Spearman’s rho = 0.657. **c** Scatter plot of shimmer. Spearman’s rho = 0.525. **d** Scatter plot of NHR. Spearman’s rho = 0.665

In the normal group, statistical analysis revealed a significant difference between the results obtained from the two software programs for NHR. No significant differences were found between the two programs for the F0, jitter, or shimmer (Table 2). However, F0 determined with MDVP showed a strong correction with that determined with Praat. The MDVP jitter data also showed a strong correlation with Praat. There was a strong correlation between MDVP and Praat regarding the NHR. Additionally, the shimmer data from Praat showed a strong correlation with MDVP (Fig. 2).

**Fig. 3:**
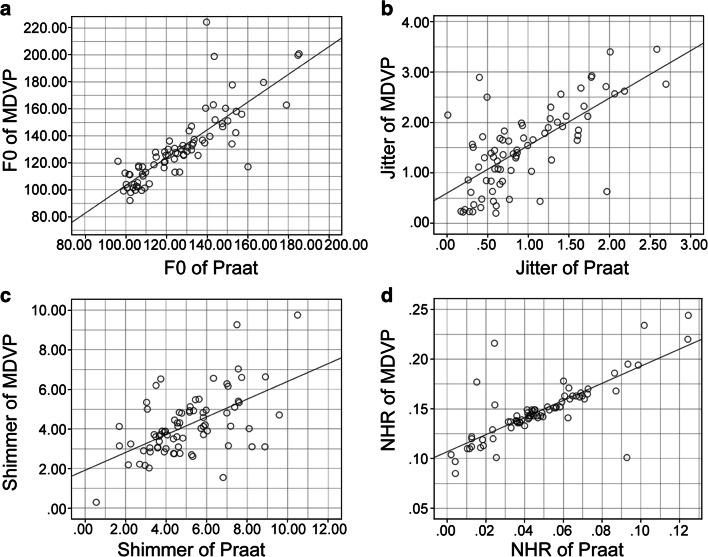
Patient voice data. The horizontal axis indicates the Praat data. The vertical axis indicates the multidimensional voice program (MDVP). **a** Scatter plot of the fundamental frequency. Spearman’s rho = 0.885. **b** Scatter plot of jitter. Spearman’s rho = 0.690. **c** Scatter plot of shimmer. Spearman’s rho = 0.562. **d** Scatter plot of NHR. Spearman’s rho = 0.772

In the OSAHS patient group, there were significant differences between the results obtained from the two software programs for F0, jitter, shimmer, and NHR. The F0, jitter, shimmer, and NHR determined with MDVP showed a strong correlation with those determined with Praat (Fig. 3).

### Consistency of MDVP and Praat measurement parameters

**Fig. 4:**
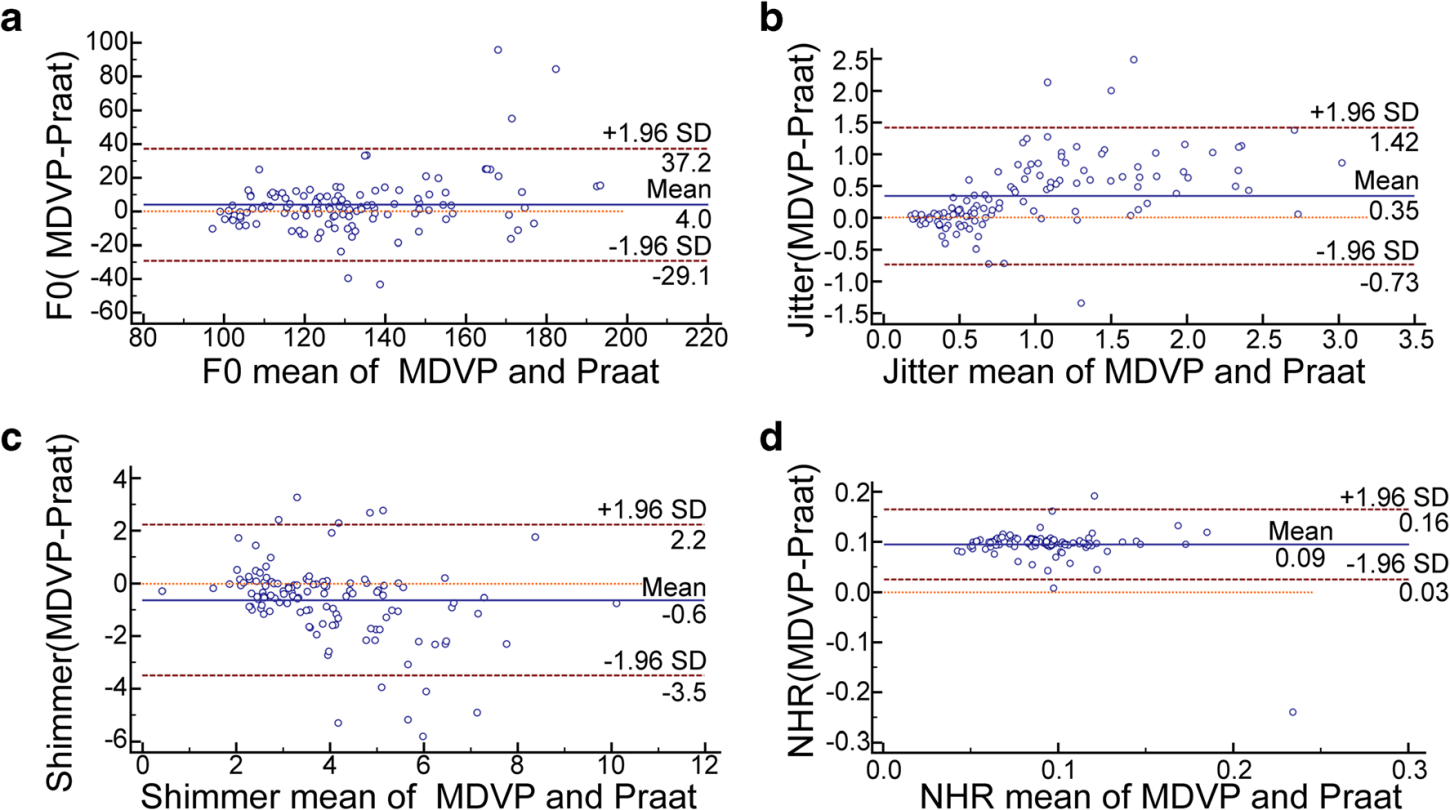
Analysis of consistency between MDVP and Praat measurements. **a** MDVP and Praat measurements of F0. **b** MDVP and Praat measurements of jitter. **c** MDVP and Praat measurements of shimmer. **d** MDVP and Praat measurements of NHR

The results of the MDVP and Praat consistency tests are shown in Fig. 4. From the figure, we concluded that there were 4.10% (5/122), 3.28% (4/122), 9.01% (11/122), and 1.63% (2/122) points outside the 95% limits of agreement (LOA) for F0, jitter, shimmer, and NHR, respectively, measured by MDVP and Praat. The 95% LoAs of F0, jitter, shimmer, and NHR are − 29.1 ~ 37.2, − 0.73 ~ 1.42, − 3.5 ~ 2.2, and 0.03 ~ 0.16, respectively. Within the 95% limits of agreement, the absolute values of the difference between the two instruments are 37.215, 1.423, 3.476, and 0.165, respectively. The average of the differences are 4.0, 0.35, − 0.6, and 0.09, respectively. From a clinical perspective, this difference is acceptable, so the two approaches are consistent.

## Discussion

It is known that the upper airway structure plays an important role in voice resonance and articulation [17]. Obstruction of any locations of the upper respiratory tract (URT) may affect the resonance and acoustic characteristics of the voice. Subramaniam et al. reported that patients with adenotonsillar hypertrophy showed significant changes in acoustic parameters compared to healthy controls [18]. On the other hand, OSAHS patients have a higher incidence of mouth breathing. Long-term mouth opening of OSAHS patients causes the inhaled airflow to be lost by the nasal cavity, causing the vocal cord surface to dry [19]. Tao et al. [20] found that with the decrease in moisture in the liquid layer on the surface of the vocal cords, the threshold voltage of the vocalization rises, and when the pressure increases to a certain extent, the vocal cords may be damaged.

This study shows that the vocal cord vibration of OSAHS patients is irregular, and the fundamental frequency perturbation, amplitude perturbation, and vibration-related acoustic parameters, such as jitter, shimmer, NHR, CQP, and CIP, are significantly higher than those of the normal group. Jitter and shimmer reflect the grade of hoarseness and roughness of the speech spectrum. NHR measures the aperiodic noise present in the analyzed signal, which can effectively reflect glottal closure. The CQP and CIP could reflect the regularity of the vocal cord vibration and the periodic change of the vocal cord contact section. A reduction in voice quality was also found in patients by using the Voice Handicap Index-10 (VHI-10).

Our study also suggests that the different software programs present different values of acoustic measures. Previous reports have shown that the MDVP and Praat analysis produce equivalent mean F0, whereas jitter and shimmer differ significantly [21–23]. However, Oguz et al. analyzed 47 normal and pathological voices with the MDVP and Praat and found no significant differences in the shimmer calculated by the software [24]. We found that in the normal group, there were no significant differences in the F0, jitter, or shimmer calculated by the two acoustic analysis programs, whereas NHR differed significantly. However, in OSAHS patients, the values of F0, jitter, shimmer, and NHR were notably different between MDVP and Praat.

The results of the study showed that MDVP appeared to differentiate the OSAHS group better than Praat. However, we also found a strong correlation between the values calculated by the two software programs. Previous studies have attempted to compare values of perturbation measures that were calculated using MDVP with those calculated using Praat. They also found a strong correlation between the values obtained by the two software programs. [22] Consistency analysis shows that MDVP and Praat have better consistency in measuring F0, jitter, shimmer, and NHR. It is possible that the values from MDVP can be linearly transformed to approximate the value calculated by Praat.

In conclusion, OSAHS patients experience long-term mouth breathing and changes in the structure of the articulation area, which result in irregular vocal cord vibration, poor glottis closure, pronunciation difficulties, hoarseness, and other vocal problems. Therefore, in clinical treatment, attention should be paid to the patient’s voice changes, and mouth-breathing habits should be corrected to avoid changes in vocal cord tissue biomechanics and vocalization due to vocal cord dehydration. The two acoustic software programs present different values of acoustic measures, and MDVP appeared to differentiate the OSAHS group better than Praat. In addition, there is a strong correlation and consistency between the values calculated by MDVP and Praat. In some cases, either of the two software programs can be used to analyze voice data.
